# Modeling maize above-ground biomass based on machine learning approaches using UAV remote-sensing data

**DOI:** 10.1186/s13007-019-0394-z

**Published:** 2019-02-04

**Authors:** Liang Han, Guijun Yang, Huayang Dai, Bo Xu, Hao Yang, Haikuan Feng, Zhenhai Li, Xiaodong Yang

**Affiliations:** 1 Key Laboratory of Quantitative Remote Sensing in Agriculture of Ministry of Agriculture, Beijing Research Center for Information Technology in Agriculture, Beijing, 100097 China; 2 College of Architecture and Geomatics Engineering, Shanxi Datong University, Datong, 037003 China; 3 National Engineering Research Center for Information Technology in Agriculture, Beijing, 100097 China; 4 College of Geoscience and Surveying Engineering, China University of Mining and Technology (Beijing), Beijing, 100083 China

**Keywords:** AGB, Machine learning, UAV, Maize, Plant height, BIOVP

## Abstract

**Background:**

Above-ground biomass (AGB) is a basic agronomic parameter for field investigation and is frequently used to indicate crop growth status, the effects of agricultural management practices, and the ability to sequester carbon above and below ground. The conventional way to obtain AGB is to use destructive sampling methods that require manual harvesting of crops, weighing, and recording, which makes large-area, long-term measurements challenging and time consuming. However, with the diversity of platforms and sensors and the improvements in spatial and spectral resolution, remote sensing is now regarded as the best technical means for monitoring and estimating AGB over large areas.

**Results:**

In this study, we used structural and spectral information provided by remote sensing from an unmanned aerial vehicle (UAV) in combination with machine learning to estimate maize biomass. Of the 14 predictor variables, six were selected to create a model by using a recursive feature elimination algorithm. Four machine-learning regression algorithms (multiple linear regression, support vector machine, artificial neural network, and random forest) were evaluated and compared to create a suitable model, following which we tested whether the two sampling methods influence the training model. To estimate the AGB of maize, we propose an improved method for extracting plant height from UAV images and a volumetric indicator (i.e., BIOVP). The results show that (1) the random forest model gave the most balanced results, with low error and a high ratio of the explained variance for both the training set and the test set. (2) BIOVP can retain the largest strength effect on the AGB estimate in four different machine learning models by using importance analysis of predictors. (3) Comparing the plant heights calculated by the three methods with manual ground-based measurements shows that the proposed method increased the ratio of the explained variance and reduced errors.

**Conclusions:**

These results lead us to conclude that the combination of machine learning with UAV remote sensing is a promising alternative for estimating AGB. This work suggests that structural and spectral information can be considered simultaneously rather than separately when estimating biophysical crop parameters.

**Electronic supplementary material:**

The online version of this article (10.1186/s13007-019-0394-z) contains supplementary material, which is available to authorized users.

## Background

Above-ground biomass (AGB) is a basic agronomic parameter for field investigation and is frequently used to indicate crop growth status, the effects of agricultural management practices, and the ability to sequester carbon above and below ground [1, 2]. The conventional way to obtain AGB is to use destructive sampling methods that require manual harvesting of crops, weighing, and recording, which makes large-area, long-term measurements challenging and time consuming. However, with the diversity of platforms and sensors and the improvements in spatial and spectral resolution, remote sensing is now regarded as the best technical means for monitoring and estimating AGB over large areas [3].

Many studies have used satellite remote-sensing images as a data source to estimate various vegetation biomasses, such as grassland [3, 4], forest [5–8], croplands [9–11], and wetland [7, 12]. Most research heretofore has focused on forest and has used the vegetation indices (VIs) to build models, especially the normalized difference vegetation index (NDVI). Although satellite remote sensing can be used for large-scale observation, it remains limited by cloud cover, satellite revisit time, coarse resolution [13]. Remote sensing using a low-altitude Unmanned Aerial Vehicle (UAV) is more flexible than satellite remote sensing, thereby overcoming these restrictions and providing remote-sensing data with higher temporal, spatial, and spectral resolution. As a result, UAV remote sensing is becoming a promising tool for frequent observations [14]. The higher spatial resolution allows more accurate extraction of plant-height information from digital images, thereby providing an attractive alternative based on modeling of plant height to estimate biomass. Plant height can be obtained from the crop surface model (CSM), which is created by using structure-from-motion techniques. Several studies have already used CSM to estimate plant height and biomass for various crops, including maize [15–17], rice [18], barley [19, 20], cotton [21, 22], sugarcane [23], wheat [24] and sorghum [16, 25]. Previous studies have confirmed that combining spectral information and plant-height information can improve biomass estimates [1, 26–29].

A literature review reveals that machine-learning methods are more prevalent in combination with satellite remote-sensing data. To estimate the biomass of a region, such approaches usually classify the vegetation first and then calculate the number of pixels of each class [30]. Yang, et al. [3] used the back propagation artificial neural network (BP-ANN) model to estimate grassland AGB at 500 m spatial resolution and demonstrated that the BP-ANN model achieves better results than the traditional multifactor regression models (R^2^ = 0.75–0.85 vs. 0.40–0.64, RMSE = 355–462 vs. 537–689 kg DW/ha). Mutanga et al. [12] used random forest regression and WorldView-2 imagery to predict wetland biomass and compared the results with those of stepwise multiple linear regression (MLR). The results demonstrate that random forest regression is more advantageous for estimating high-density biomass. Zhang et al. [31] used Landsat data and four machine-learning regression algorithms [support vector machine (SVM), random forest (RF), k-nearest neighbor (k-NN), and ANN] to estimate both live and total sawgrass biomass. The results indicate that ANN and SVM produce similar results for estimating live biomass.

However, few studies have used structural and spectral information provided by UAV remote sensing in combination with machine learning to estimate maize biomass. The specific objectives of this study therefore include (1) comparing the performance of different machine-learning modeling methods to estimate maize AGB, (2) verifying an improved method to extract plant height and obtain an indicator to estimate AGB; and (3) to explore the potential of machine-learning modeling based on remote sensing to quantify AGB.

## Methods

### Experimental materials and field measurements

**Fig. 1:**
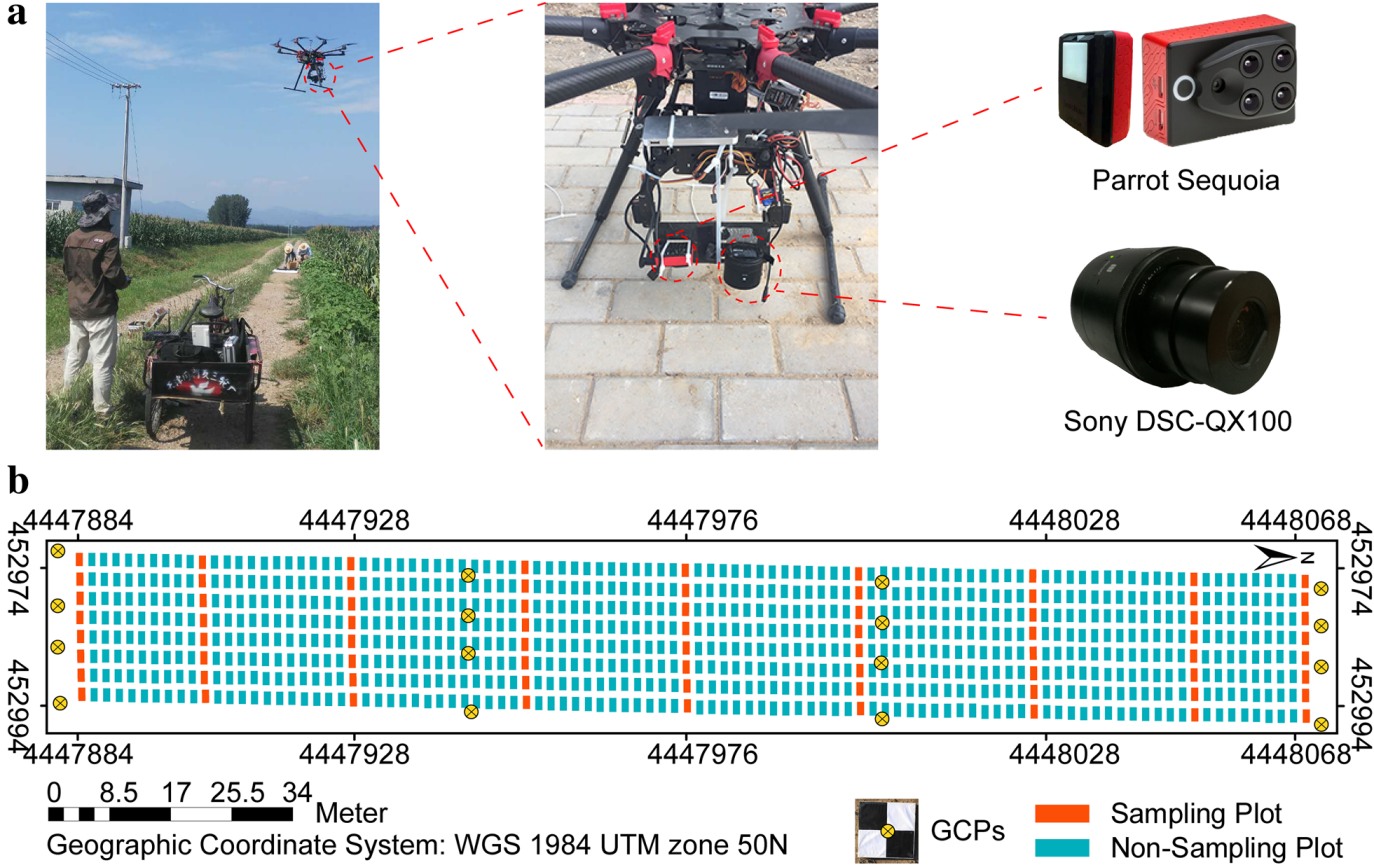
Maize experiment at Xiao Tangshan National Precision Agriculture Research Center, Changping, 2017. **a** UAV platform and sensors. **b** Experimental site. “GCPs” refers to the ground control points used to limit errors and improve the accuracy of plant-height extraction

The study area was located in the research station of Xiao Tangshan National Precision Agriculture Research Center of China, Changping District of Beijing City (115°50′17″–116°29′49″E, 40°20′18″–40°23′13″N), at an average elevation of 36 m. The study area has a warm temperate semi-humid continental monsoon climate, with the rainy season lasting from June to August. The average annual temperature is 11.8 °C [29]. Eight hundred plots were planted at a seeding density of 6 plants/m^2^ with a row spacing of 0.6 m and divided into four groups: mixed, TEM (temperate), TST (tropical/subtropical) and DH (doubled-haploid) according to the genetic background differences. The plots were 2 m × 2.4 m, and 72 plots were used as sampling plots for destructive biomass measurements; all other non-destructive measurements were made on other plots on June 28 and July 11, 2017 (Fig. 1). All plots were seeded on May 15, 2017.

Sixteen ground control points (GCPs) distributed evenly within the field were used to obtain accurate geographical references and were located with millimeter accuracy by using a Differential Global Positioning System (DGPS, South Surveying & Mapping Instrument Co., Ltd., Guangzhou, China). Three plants were selected at random in the central part of sampling plots for measuring plant height and fresh biomass. The plant height was measured manually with a telescopic leveling rod. The mean height of the three plants was used as the canopy height of the given sampling plot. Next, the three plants were subjected to destructive biomass sampling. Fresh biomass was sealed in plastic bags and weighed on the same day. Finally, the masses were rescaled to kg/m^2^ by counting the actual number of plants in each sampling plot. Because 14 fresh biomass samples were not available due to a record problem in the laboratory, the total fresh biomass sample size numbers 130. Table 1 summarizes the data obtained from field measurements.

**Table 1:** Basic statistics of the field measurements

Date	Object	Min	Max	Mean	CV (%)
June 28, 2017	PHobs (cm)	57.6	109	81.1	14.4
	AGB (kg/m^2^)	0.30	5.02	2.39	39.71
July 11, 2017	PHobs (cm)	66	213	149.9	17.4
	AGB (kg/m^2^)	0.51	3.68	2.07	29.05

PHobs, plant height measured by manpower. CV, coefficient variation is used to describe the central tendency and dispersion of data, which is calculated with mean and standard error

### Unmanned aerial vehicle and camera setup

The digital and multispectral imagery was collected over three flights with an octocopter DJI Spreading Wings S1000 UAV (SZ DJI Technology Co., Shenzhen, China) platform equipped with two cameras. Digital imagery was collected by using a 20.2 megapixel Cyber-shot DSC-QX100 (Sony Electronics, Inc., Tokyo, Japan). Multispectral imagery were collected with a 1.2 megapixel Parrot Sequoia camera (MicaSense Inc., Seattle, USA), which captures four discrete spectral bands: green (wavelength = 550 nm, bandwidth = 40 nm), red (660 nm, 40 nm), red-edge (735 nm, 10 nm), and near infrared (790 nm, 40 nm). The radiometric calibration images of Parrot Sequoia camera were captured on the ground before and after each flight by using a calibrated reflectance panel (MicaSense Inc., Seattle, USA). The Parrot Sequoia camera relies on a sunshine sensor to automatically adjust the readings to ambient light to minimize error during image capture [32].

Flight paths over the trial area were designed by the DJI ground station, yielding six strips. The forward overlap was 80% and the lateral overlap was 75%. The flight speed was fixed at 6 m/s. ISO and shutter speed were fixed at 160 and 1/2000, respectively. The flight altitude above ground level (AGL) on June 28 and July 11, 2017 was 60 m. The ground sampling distances for digital and multispectral imagery were approximately 1.3 and 5.5 cm, respectively. To obtain a high-precision digital elevation model, the flight altitude above ground level for the first flight on June 8, 2017 was 40 m, yielding a ground sampling distance of 0.72 cm. The details of the UAV data acquisition are listed in Table 2.

**Table 2:** Details of UAV data acquisition

Flight	Camera	Imagery quantity	GSD (cm)	AGL (m)
1	Digital	120	0.72	40
2	Digital	94	1.33	60
	Multispectral	110 per band	5.53	
3	Digital	91	1.35	60
	Multispectral	105 per band	5.54	

### Image processing and data extraction

A Pix4Dmapper Pro (version 4.0, PIX4D, Lausanne, Switzerland) was used to produce digital surface models (DSMs), generate orthomosaics, do radiometric calibration, and calculate vegetation indices. The key steps of this process included image geolocation, importing ground control points, aligning images, building a dense point cloud, building a DSM and an orthomosaic, processing and calibrating radiometric information, and generating vegetation indices (VIs) maps. Sixteen ground control points in the Pix4D project were used to georeference the study area, increase the global accuracy, and reduce noise. The contents listed in Table 3 were used to evaluate the accuracy of DSMs. Radiometric calibration was done by using radiometric calibration images with known reflectance values provided by MicaSense. The radiometric corrections were used to improve the radiometric quality of the data and correct the images reflectance. Seven near-infrared VIs maps and four visible-band VIs maps were produced by using the index calculator in the Pix4D software. The calculated VIs are listed in Table 4. Related computation formulas are shown in Additional file 1.

**Table 3:** Processing quality report for evaluating the accuracy of DSMs

Flight	X error (cm)	Y error (cm)	Z error (cm)	Total error (cm)	Point density (points/cm^2^)
1	0.93	1.02	0.49	1.46	47.9
2	1.15	1.72	0.64	2.17	14.2
3	1.28	1.46	0.73	2.08	13.7

Error represents the root mean square error (RMSE) of the GCPs. X represents longitude; Y represents latitude; Z represents altitude

**Table 4:** Spectral vegetation indices used in this study to evaluate maize above ground biomass

VIs	Formula	Application	References
CIgreen	$\frac{NIR}{GREEN} - 1$	Chlorophyll	[33]
CIrededge	$\frac{NIR}{REDEDGE} - 1$	Chlorophyll	[33]
CVI	$NIR * \frac{RED}{{GREEN^{2} }}$	Chlorophyll	[34]
NDRE	$\frac{NIR - REDEDGE}{NIR + REDEDGE}$	Chlorophyll	[35]
RVI	$\frac{NIR}{RED}$	LAI, biomass	[36–38]
NDVI	$\frac{NIR - RED}{NIR + RED}$	Biomass	[39, 40]
WDRVI	$\frac{0.1NIR - RED}{0.1NIR + RED}$	LAI, biomass	[41]
GLI	$\frac{2 * GREEN - RED - BLUE}{2 * GREEN + RED + BLUE}$	Chlorophyll	[42, 43]
VARI	$\frac{GREEN - RED}{GREEN + RED - BLUE}$	LAI, biomass	[44, 45]
ExG	$2 * GREEN - RED - BLUE$	Canopy coverage	[46]
NGRDI	$\frac{GREEN - RED}{GREEN + RED}$	Canopy coverage	[39]

In the second column, the letters represent spectral reflectance, such as NIR, which represents near-infrared reflectance in the UAV multispectral images.

Because these VIs can respond to different targets, we used Otsu algorithm [47] to determine thresholds and binarize the VIs maps, and then separated plants from the soil background in these VIs maps. ArcMap (version 10.2, Esri Inc., Redlands, USA) was used to create the area of interest (AOI) with separated plant areas and to extract the average VI for each plot. This process was also applied to extract plant height.

The CSM, which is widely used to extract plant-height information from different crops, was used in the present study. The CSM can be obtained by subtracting the digital elevation model from the DSM by using the raster calculator in ArcMap. On June 8th, 2017, the maize was about at the growth stage 13 (BBCH-scale) [48] and had an average height of less than 20 cm. We extracted 1332 elevation points from the DSM on June 8th from locations not covered with vegetation and interpolated a digital elevation model (DEM) from these data by using the Kriging spatial interpolation method. Thus, two CSMs were created (one on June 28th and one on July 11th).

We propose an improved method to filter out the point cloud formed by the soil background and the lower leaves. The method involves using image segmentation and kernel neighborhood maximal calculation (i.e., kernel thinning) to create a set of pixels that image the upper leaves of multiple plants. Resampling was used to control the number of pixel points involved in computation. These pixel points have three-dimensional spatial coordinates and thus have spatial distribution characteristics. Considering spatial variation, Kriging interpolation was done on these three-dimensional pixel points to generate a plant-height surface. The peak values on the surface were extracted as the representative values of plant height at the plot scale. Using areas of interest (only cover vegetation), we extracted plant-height information from the above results by using ENVI software (version 4.5, Esri Inc., Redlands, USA). All the concepts and terminology related to the above contents are illustrated in Additional file 2.

The canopy elevation relief ratio (CRR) were calculated by using the plant-height data. The CRR is commonly used in forestry studies as a metric that describes the relative shape of the canopy; it reflects the degree to which outer-canopy surfaces are in the upper (CRR > 0.5) or lower (CRR < 0.5) portions of the height range [49, 50]. Because the CRR is susceptible to outliers, we made a simple adjustment. The BIOVP is the sum of pixel values (i.e., plant height) in the CSM without soil background and after resampling (Additional file 2). The definitions of these three variables appear in Table 5.

**Table 5:** Definitions of three plant height-related metrics this study used for biomass estimation

Variable	Formula (method)	Description	References
CRR	${\text{CRR}} = \frac{{PH_{{mean}} - PH_{{10\% {\text{min}}}} }}{{PH_{{10\% {\text{max}}}} - PH_{{10\% {\text{min}}}} }}$	Metric to describe the relative shape of the canopy in forestry studies	[19, 20]
PHkri	kernel thinning and Kriging spatial interpolation	Plant-height metric	
BIOVP	${\text{BIOVP}} = \sum\nolimits_{{\text{i}}}^{{\text{N}}} {S*PH_{{\text{i}}} }$	Volume metric to estimate crop biomass within certain spatial ranges	

CRR is the canopy elevation relief ratio with a simple adjustment. The maximum (PH_10%max_) and minimum (PH_10%min_) values are calculated by using the top 10% and bottom 10% plant-height data in a plot, respectively. PHkri is the plant height calculated by using a Kriging interpolation. BIOVP is a volume metric used to estimate crop biomass within a plot. *S* represents the area covered by plants after resampling and image segmentation, *PH*_i_ indicates the plant height represented by the *i*th pixel, and *N* is the number of pixels within *S*.

### Selecting predictor variables

**Fig. 2:**
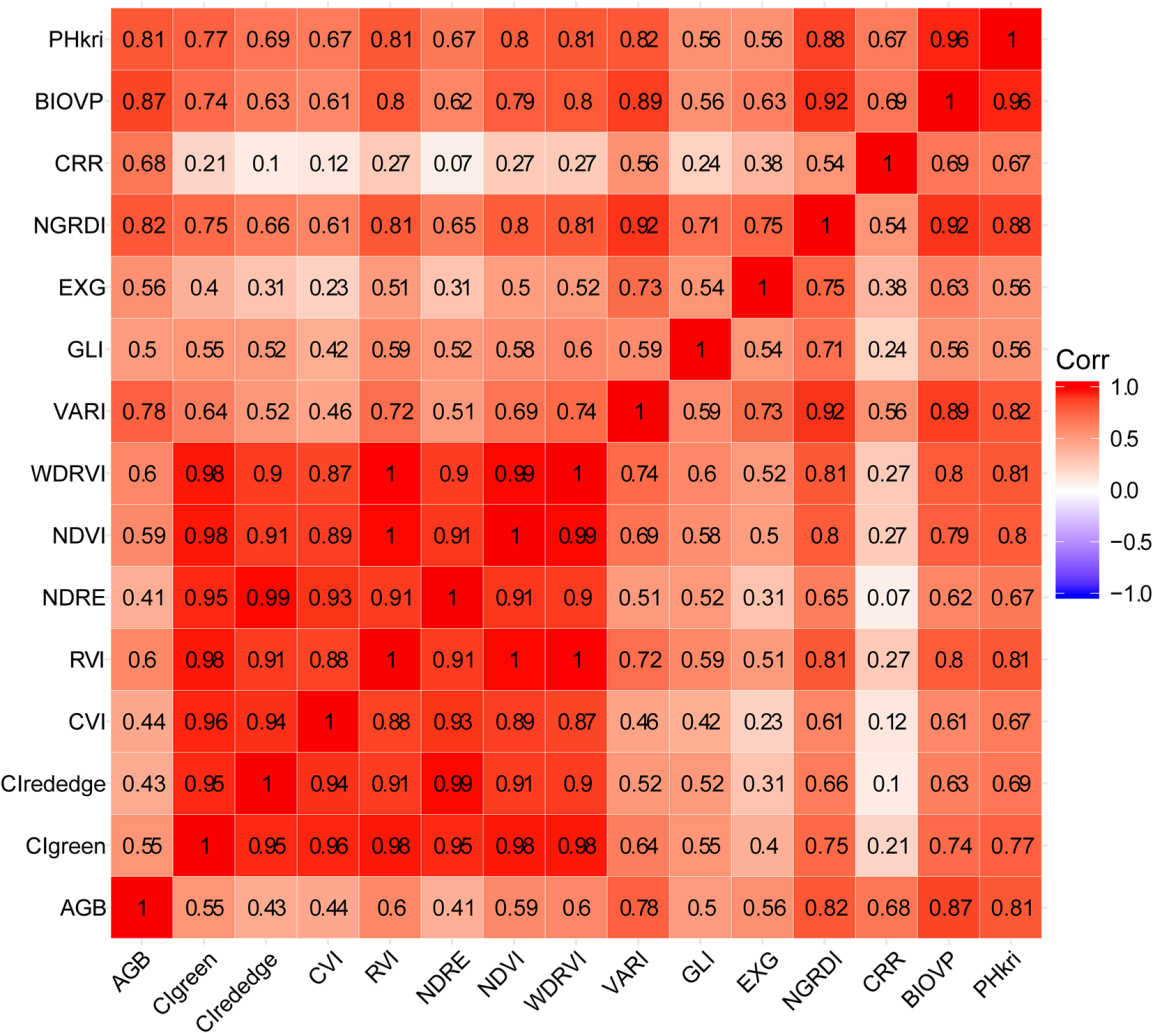
Pearson’s correlation among predictor variables

A high Pearson’s correlation was found between AGB and some predictors, such as BIOVP, PHkri, VARI, CRR, and NGRDI. However, multi-collinearity is also present between these continuous predictor variables (Fig. 2). Data redundancy and multi-collinearity can increase model complexity and seriously affect regression performance [51]. The goal of selecting predictor variables is to find the optimal subset from the input, thereby reducing the effect of noise or uncorrelated variables, improving prediction performance, and reducing runtime [52, 53]. The recursive-feature-elimination (RFE) algorithm provides a way to automatically select predicator variables by repeatedly creating a model and removing predictors with low weights. This study uses the R package “caret” (version 6.0-80) [54] to implement this algorithm, which is based on the Gini criterion with repeated tenfold cross validation within the context of a random forest model [55, 56]. The subset of the recursive results with the smallest error served as the subset of predictors. The importance of the selected predictor variables was quantified based on the percent increase in mean square error (IncMSE%) and total increase in node purities (IncNodePurity) [28, 57].

### Modeling and resampling

To obtain the most suitable model for estimating maize AGB by comparative analysis, we adopted three modeling strategies and created four models (Table 6). SVM and ANN models are strict in requiring predictor variables with a common scale, so data pre-processing techniques should be performed on the training set before modeling [57]. In this study, these pre-processing techniques contained data standardization and skewness transformations.

**Table 6:** Modeling strategies and methods implemented in this study

Model	Strategy	Method	Tuning parameters
MLR	Linear regression	Multiple linear regression	–
SVM	Nonlinear regression	Radial basis function SVM	*Sigma* and *C*
ANN	Nonlinear regression	Averaged artificial neural networks	*H* and *lambda*
RF	Regression trees	Random forest	*mtry*

The cost-penalty parameter *C* indicates the tolerance to error. When *C* is large, the model cannot tolerate large error and becomes more flexible, which leads to overfitting. When *C* is small, the model becomes rigid and is more prone to underfitting. *Sigma* is a parameter of the radial basis function: a smaller *sigma* corresponds to fewer support vectors, which affects model training and prediction accuracy [58]. *H* is the number of hidden units that are linear combinations of some or all predictors, *lambda* is weight decay that restricts overfitting, and *mtry* is the number of randomly selected predictors at each split.

Because the 130 samples were each composed of two subsamples corresponding the two different observation dates, the stratified random sampling method was used to divide total samples into training set and test set with a split ratio of 70:30. The tenfold-repeated cross-validation resampling method was used to train and tune models. In this method, the training set was partitioned randomly into 10 subsets of approximately equal size. Each time, 90% of all samples was used to fit the model and the remaining 10% was used as a test set to estimate performance metrics. The 10 resampled performance estimates (i.e., the evaluation metrics of the model’s predictive capabilities) were summarized to analyze the relationship between the tuning parameters and model utility. For one modeling strategy, this procedure was repeated 10 times, yielding 10 random partitions of the training set and 100 training models. With the exception of the MLR model, each model had at least one tuning parameter. The grid-search method with a set of greedy search parameters was applied to find the optimal parameters [59].

To examine how the resampling method affected the training model, a modified bootstrap resampling (632 + boostrap) [60] method was used for comparison. This method consists of repeatedly and randomly selecting a sample from the training set.

Three types of regression diagnostics plots were used to check if the model works well for samples. The tuning-parameter plot shows how to determine the optimal parameter configuration when retaining an evaluation metric during the resampling procedure. The plot of observed values versus predicated values shows outliers or areas where the model is not calibrated and allows us to assess the proximity of the predictions to the actual values. The plot of residual values versus predicated values allows us to check whether a phenomenon appears with a different variance. If the plot shows that residuals do not appear to be randomly scattered about zero with respect to the predicted values, major predictors may be missing from the model. In this plot, marginal rugs were used to visualize the distribution of data with respect to each axis [61].

The coefficient of determination (R^2^), RMSE, and mean absolute error (MAE) were used as evaluation metrics to quantify the performance of the regression model and to determine how well the model predicts new data and whether the model is too complicated. Equations (1)–(3) are used to calculate R^2^, MAE, and RMSE:

1 $${\text{R}}^{2} = 1 - \frac{{\sum\nolimits_{i = 1}^{N} {\left( {y_{i} - \hat{y}_{i} } \right)^{2} } }}{{\sum\nolimits_{i = 1}^{N} {\left( {y_{i} - \bar{y}_{i} } \right)^{2} } }}$$

2 $${\text{MAE}} = \frac{1}{N}\sum\limits_{i = 1}^{N} {\left| {y_{i} - \hat{y}_{i} } \right|}$$

3 $${\text{RMSE}} = \sqrt {\frac{1}{N}\sum\limits_{i = 1}^{N} {\left( {y_{i} - \hat{y}_{i} } \right)^{2} } }$$

where *N* is the total sample size, $y_{i}$ is the *i*th measured AGB of the sample, $\hat{y}_{i}$ is the *i*th predicted value, and $\bar{y}_{i}$ is the *i*th mean measured value.

Comparison analysis was done for both the training set (during cross validation) and the test set. Random-number seeds were set before training each model to ensure that each model had the same data partition and repeats. The results included evaluation metrics from the final model, and we applied a statistical hypothesis to check whether a statistically significant difference existed in the results. More specifically, the student T test was applied if the results were normally distributed and the Wilcoxon rank sum test was applied if the distribution was unknown. The importance to a model of the various predictor variables was evaluated by changing the input value and comparing the sensitivity of the output of the training model, and importance scores are scaled to have a maximum value of 100 and a minimum value of 0.

**Fig. 3:**
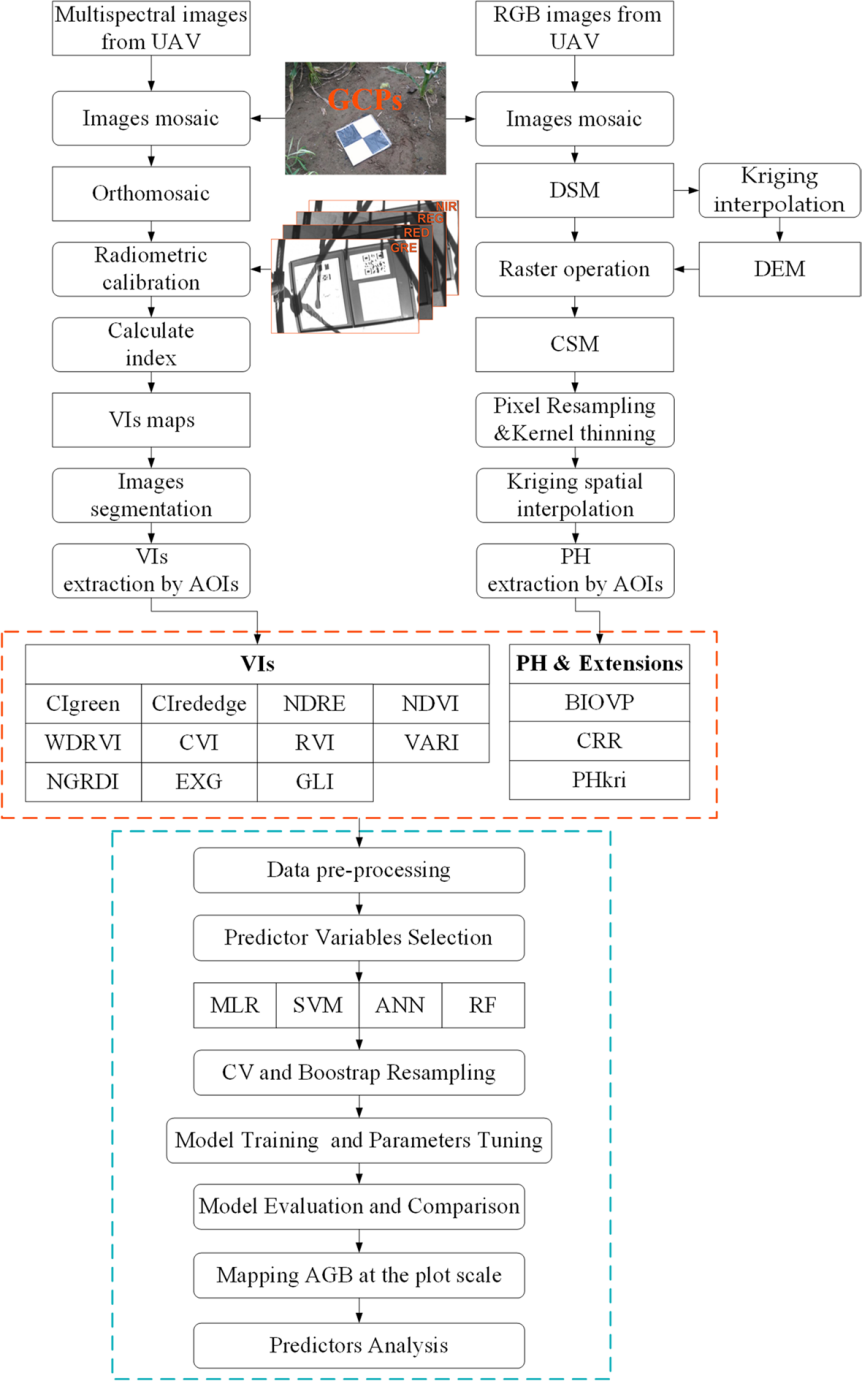
Schematic diagram of methodology used in this study. The red rectangular box contains all predictors extracted from the UAV images, and the blue rectangular box contains modeling methods and analysis procedures

The Caret package was used to create these machine-learning models in R (version 3.5.1, R Development Core Team, 2018), which created a comprehensive framework for building and evaluating predictive models [57]. The R package ggplot2 and its extension were used to draw figures. A schematic diagram of the methodology appears in Fig. 3. Relevant R code were shown in Additional file 3.

## Results

### Model evaluation and comparison

**Fig. 4:**
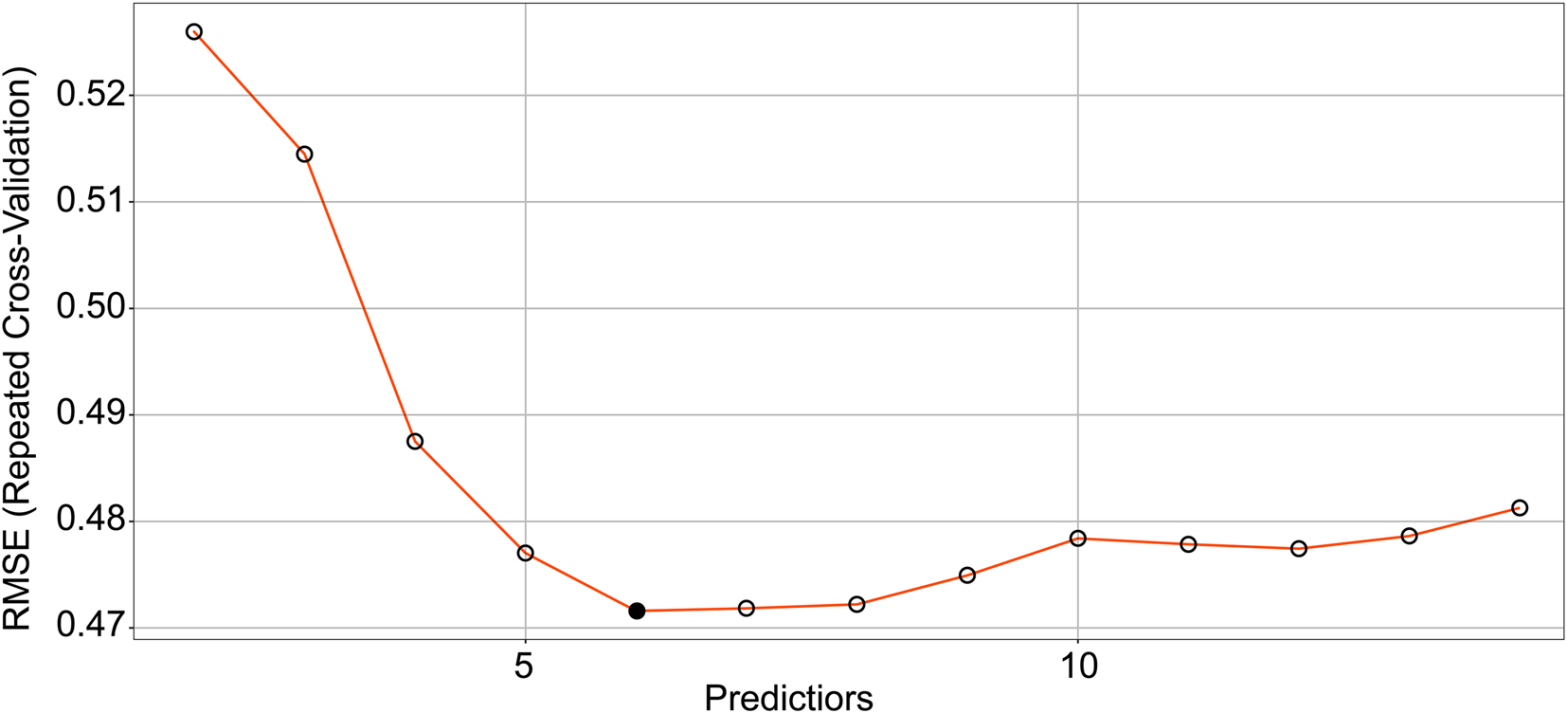
Cross-validated RMSE by recursive feature elimination (RFE) algorithm. RMSE is minimized with six predictor variables

**Fig. 5:**
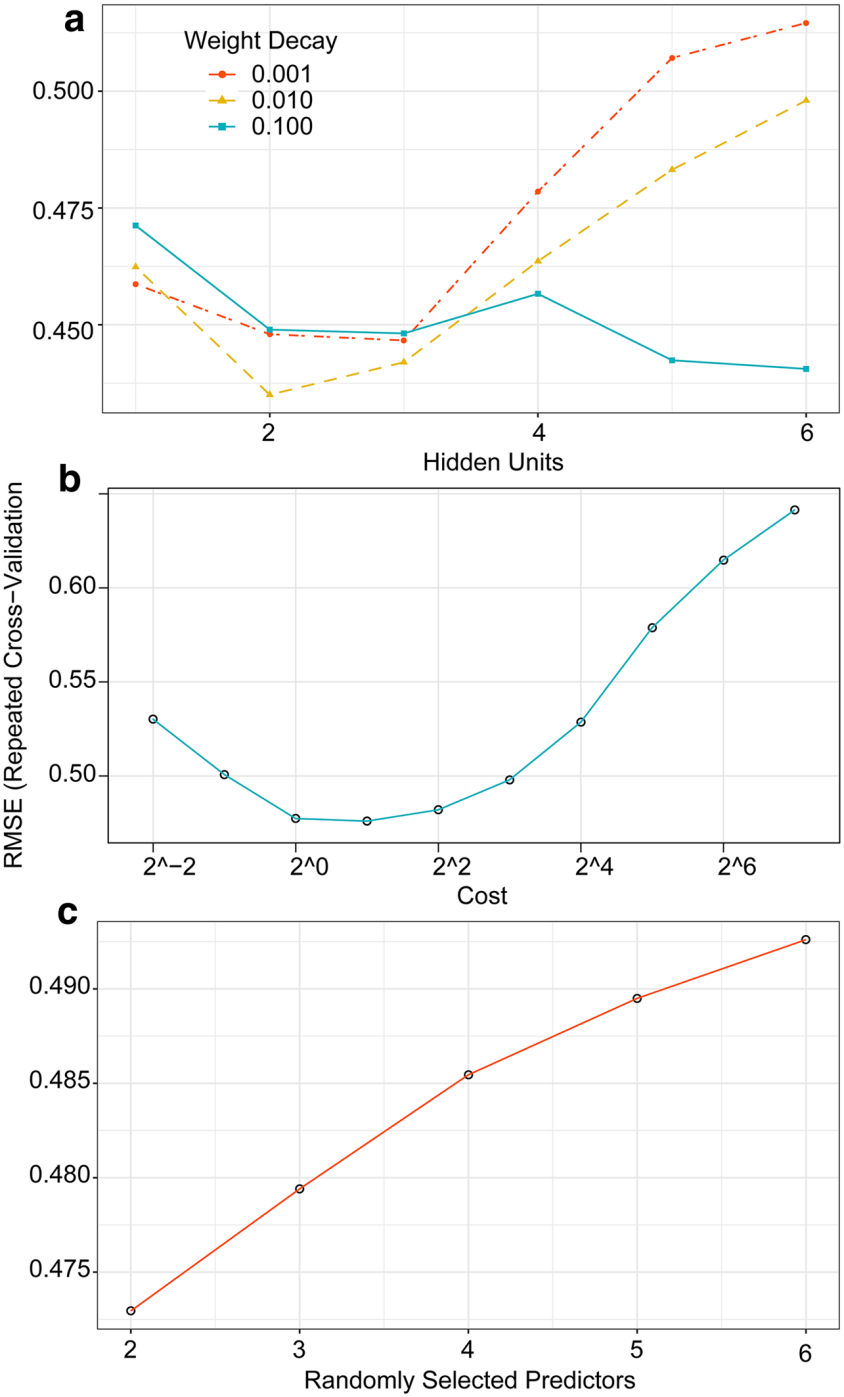
Tuning parameters when using grid-search method and cross validate. The RMSE was used to select the optimal model using the smallest value. **a** Artificial neural network model. The optimal ANN model used a medium degree of regularization (i.e., *lambda* = 0.01) and a single hidden layer with two hidden units. **b** Support vector machine model. When using the radial basis function, the SVM model was numerically optimal at *sigma* = 0.3592 and *C* = 2 on the log_2_ scale. **c** Random forest model. The RF model was numerically optimal at *mtry* = 2

Repeated cross validation was used to determine the optimal number of predicator variables required to minimize the RMSE. Figure 4 shows that the RFE algorithm found a minimum RMSE (0.472) where a subset contained six predictor variables. Sorted in terms of decreasing importance of variables, these selected predictors were BIOVP, PHkri, NGRDI, VARI, CRR, and NDVI, which were used for training models and obtaining optimal parameters. For the ANN model, three different weight-decay values were evaluated (*lambda* = 0.001, 0.01, and 0.10) along with a single hidden layer with sizes ranging from one to six hidden units. The optimal model was the average of five different neural networks created by using different initial values for parameters and used two hidden units with a medium degree of regularization (i.e., *lambda* = 0.01). For the SVM model, the kernel parameter was estimated analytically to be *sigma* = 0.3592 and the model was tuned over ten cost values between 0.25 and 128 on the log_2_ scale. A cost parameter *C* = 2 for the optimal model minimized the RMSE. As the cost parameter continued to increase, the error also began to increase and the model underfit. The RF model was numerically optimal at *mtry* = 2, which is also a recommended value (i.e., one third of the number of predictors) [62]. Figure 5 shows how to use the grid-search method to evaluate the optimal parameters of these models.

**Fig. 6:**
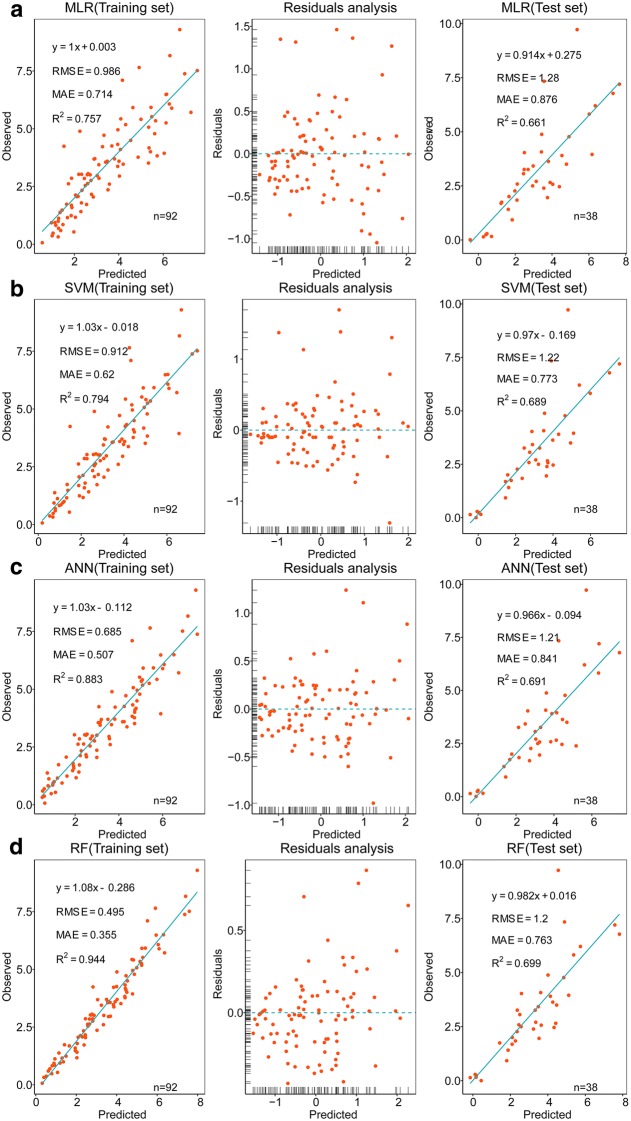
Regression diagnostics plots based on four modeling methods. **a** Multiple linear regression model. **b** Support vector machine model. **c** Artificial neural network model. **d** Random forest model. The horizontal axis represents the predicted AGB obtained from the model, and the vertical axis represents the AGB measured manually at ground level. Marginal rugs in the residuals-analysis plot were used to visualize the distribution of data on each axis. The solid cyan line represents a 1:1 relationship. In the training set, the four models tended to underestimate the AGB, whereas in the test set they tended to overestimate the AGB

Regression diagnostic plots with marginal rugs from four models all showed that the distribution between the predicted values and the residuals appears to be random about zero, which infers that the six selected variables can adequately replace the other variables (Fig. 6). The RF model had the narrowest residual interval, whereas the MLR model had the widest residual interval. In the training set, the RF model was the most accurate of the four models, with R^2^ = 0.944 (RMSE = 0.495, MAE = 0.355). Overall, the nonlinear model performed significantly better than the linear model (i.e., the MLR model, with RMSE = 0.986, MAE = 0.714, R^2^ = 0.757), which revealed a nonlinear relationship between response variable (i.e., AGB) and predictors. Compared with the training set, the prediction ability of the four models in the test set was, to varying degrees, worse. This result may be caused by the small sample size of the test set, which leads to unstable results. The ANN and RF models had a larger R^2^ (i.e., 0.691 and 0.699, respectively) and a smaller RMSE (i.e., 1.210 and 1.200, respectively) with the test set, and so were better than other models. The two outliers located in the upper-right corner occurred with the test set and may be due to measurement errors. Therefore, poor-quality data was also one reason for the degraded performance of the model with the test set.

**Fig. 7:**
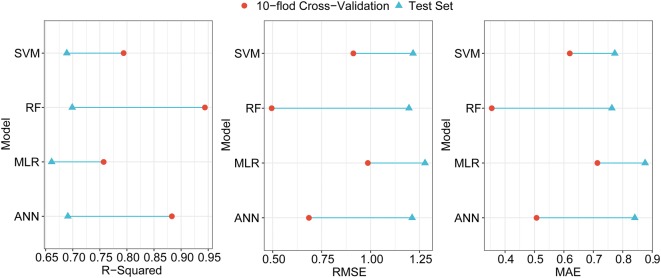
Difference between performance metrics calculated by using cross-validation and test-set data. Longer lines represent a larger performance difference. ANN and RF models had a higher R^2^ and a lower RMSE both in the training set and test set, which indicates that they performed better than other models

Figure 7 shows the difference between performance metrics calculated by using the training-set data and by using the test-set data. Longer lines represented a larger performance difference between the two sets of data. Random forest model was not sensitive to outliers, so it performed best in training set with a relatively large sample size. However, the performance advantages of random forest model in test set with a small sample size are not fully demonstrated in this study. In other words, proportion and distribution of outliers and small sample size narrowed the performance difference between models in the test set, because the advantages and disadvantages of the model were not fully exposed. Overall, the RF model performed best with both the training set and the test set and was thus selected for this study to produce the AGB maps for June 28 and July 11, 2017.

**Fig. 8:**
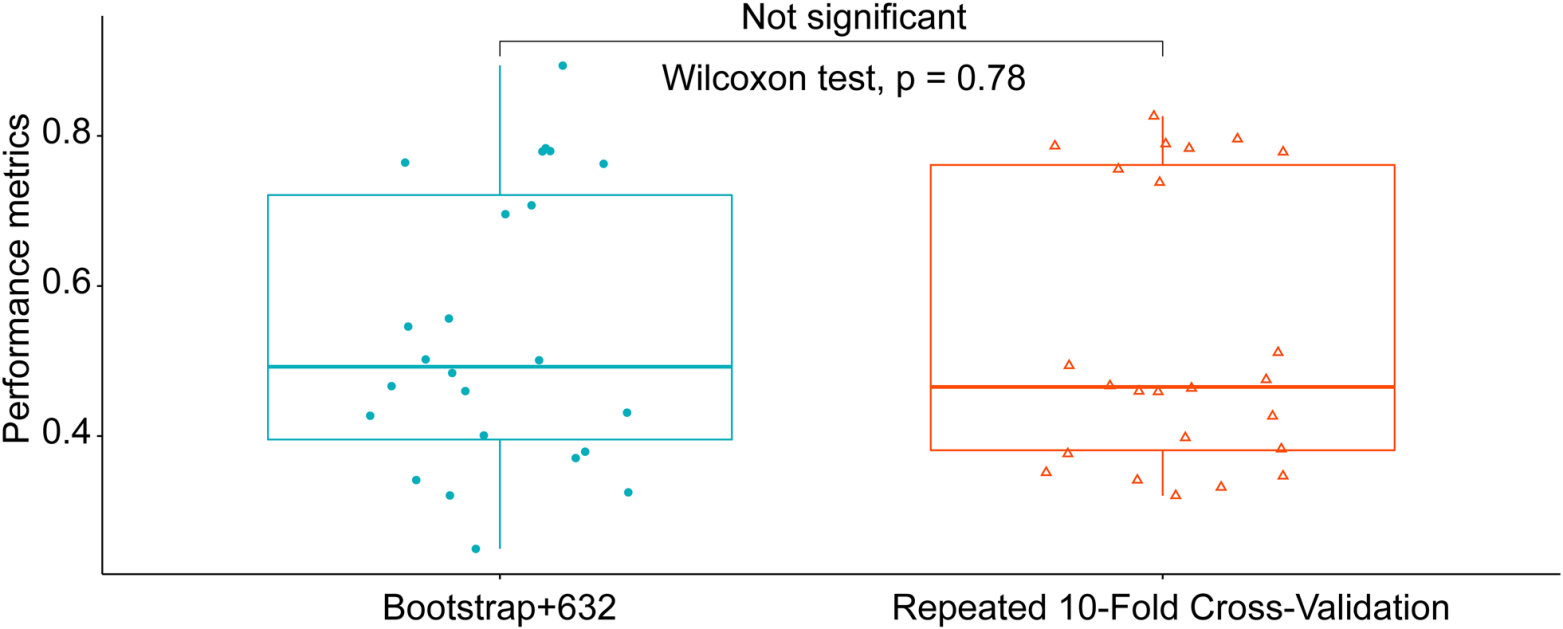
Test of significant difference of two resampling methods. For a p value > 0.05, the Wilcoxon test accepted the null hypothesis that two sets of performance metrics calculated by using two different resampling methods were drawn from the same distribution

At the 5% significance level, the Wilcoxon test accepted the null hypothesis that two sets of performance metrics calculated by using two different resampling methods were drawn from the same distribution (Fig. 8). From this we inferred that the two resampling methods have no significant difference on creating the optimal model.

### Mapping above-ground biomass of maize

**Fig. 9:**
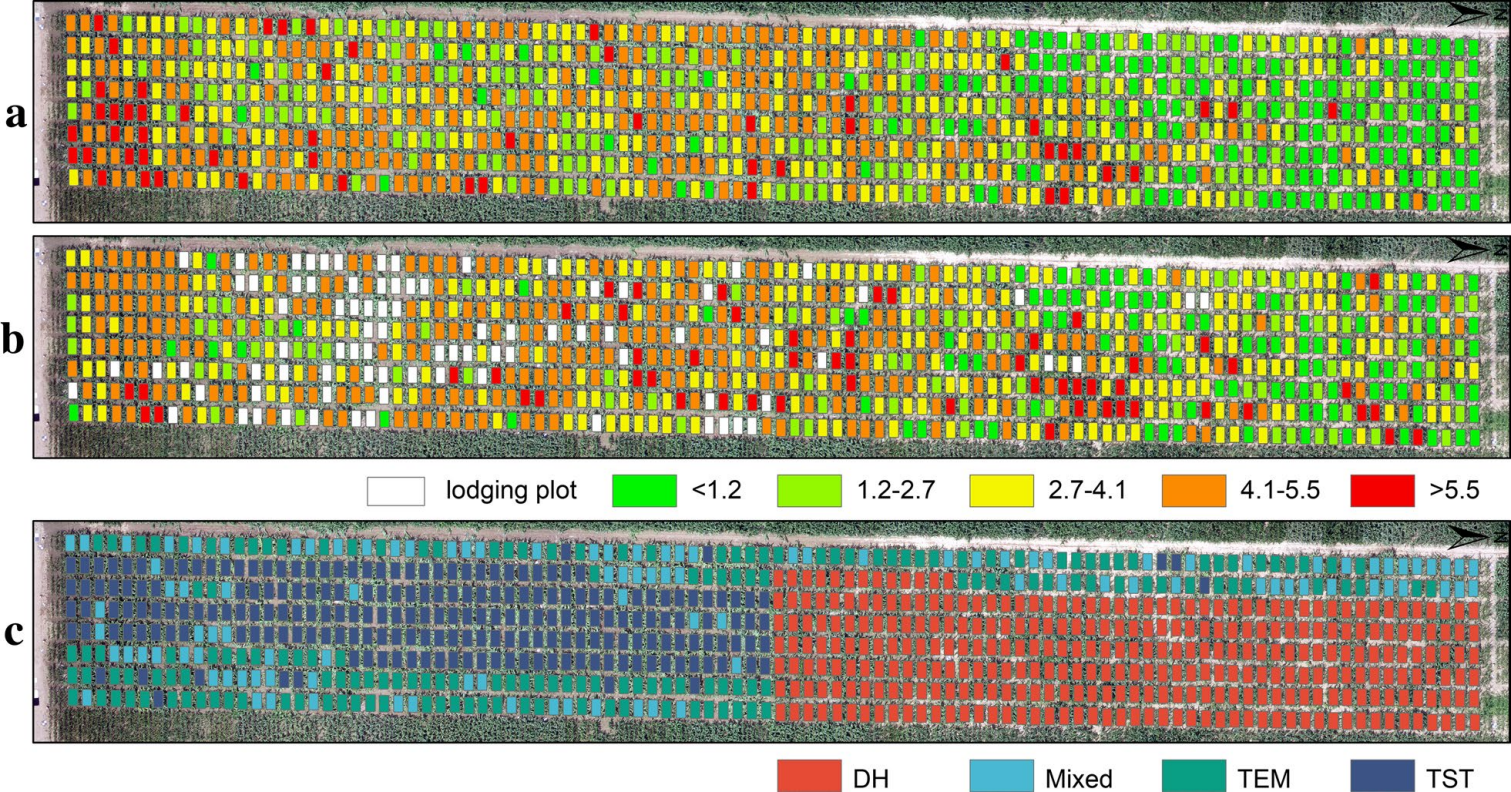
Spatial distribution of maize AGB (kg/m^2^) at the plot scale from RF model estimation. **a** On June 28, 2017. **b** On July 11, 2017. **c** Distribution of maize plots with four genetic backgrounds

We estimated the spatial distribution of the AGB at the plot scale based on the selected RF model (Fig. 9a, b). During the period from June 28 to July 11, 2017 strong winds and heavy rainfall lodged maize in some plots, which resulted in abnormal fluctuations in both plant height and spectral information. This was the main reason that the predicted values of some plots decreased instead of increasing (Fig. 9b). In some plots, maize grew rapidly because of abundant rain, causing the AGB to increase significantly in the short term. Most of the lodging plots were planted with TST group (Fig. 9c). Han et al. [63] provides in-depth analysis of the underlying association between maize lodging and the selected feature factors in this study area. These factors included but not limited to genetic backgrounds, terrain and plant height.

**Fig. 10:**
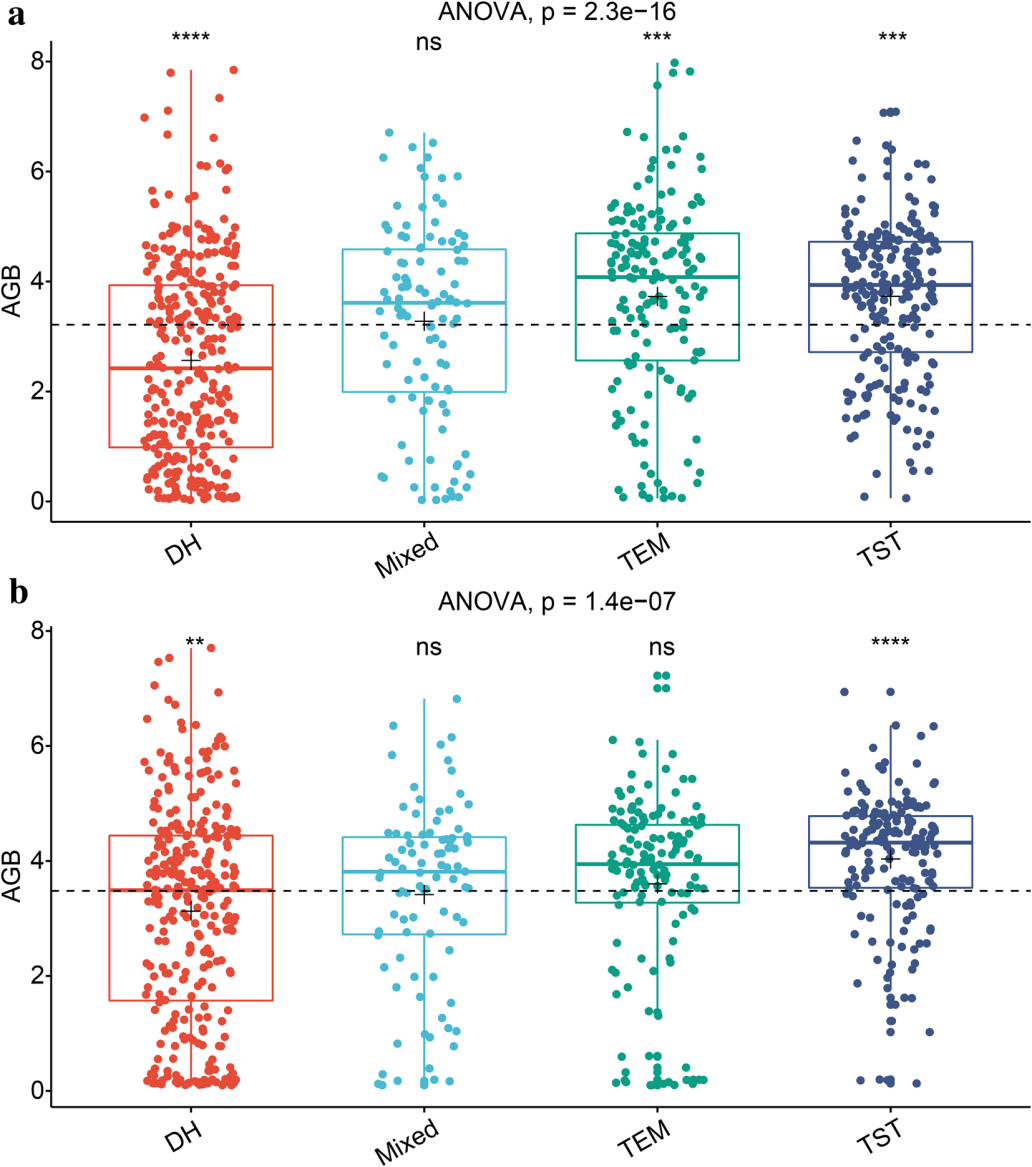
Genotypic differences in maize AGB (kg/m^2^). **a** On June 28, 2017. **b** On July 11, 2017. DH, Mixed, TEM and TST represent four genetic backgrounds of maize. The dashed black line indicates the mean biomass from all plots (i.e., baseMean). The black plus sign indicates the mean biomass from each genetic background (i.e., group). ANOVA is used to determine the existence of differences among four-group means. The Wilcoxon signed-rank test is used to perform comparison of each group against all without grouping (i.e., baseMean). The following convention for symbols indicates statistical significance: p > 0.05 (ns); p ≤ 0.05 (*); p ≤ 0.01 (**); p ≤ 0.001 (***); p ≤ 0.0001 (****). When the test is significant, it is found that DH group is significantly low-AGB compared to all and TST group is high-AGB compared to all. Because the test is not significant, there is no significant difference in the AGB between Mixed group and all

ANOVA overall had a *p* value < 0.05, so we further compared the differences in the mean AGB between each group and all plots without grouping. When Wilcoxon signed-rank test was significant, it was found that DH group was significantly low-AGB compared to all (i.e., without grouping) and TST group was high-AGB compared to all. Because the test was not significant, there was no significant difference in the AGB between Mixed group and all (Fig. 10).

### Importance of predictors and BIOVP

**Fig. 11:**
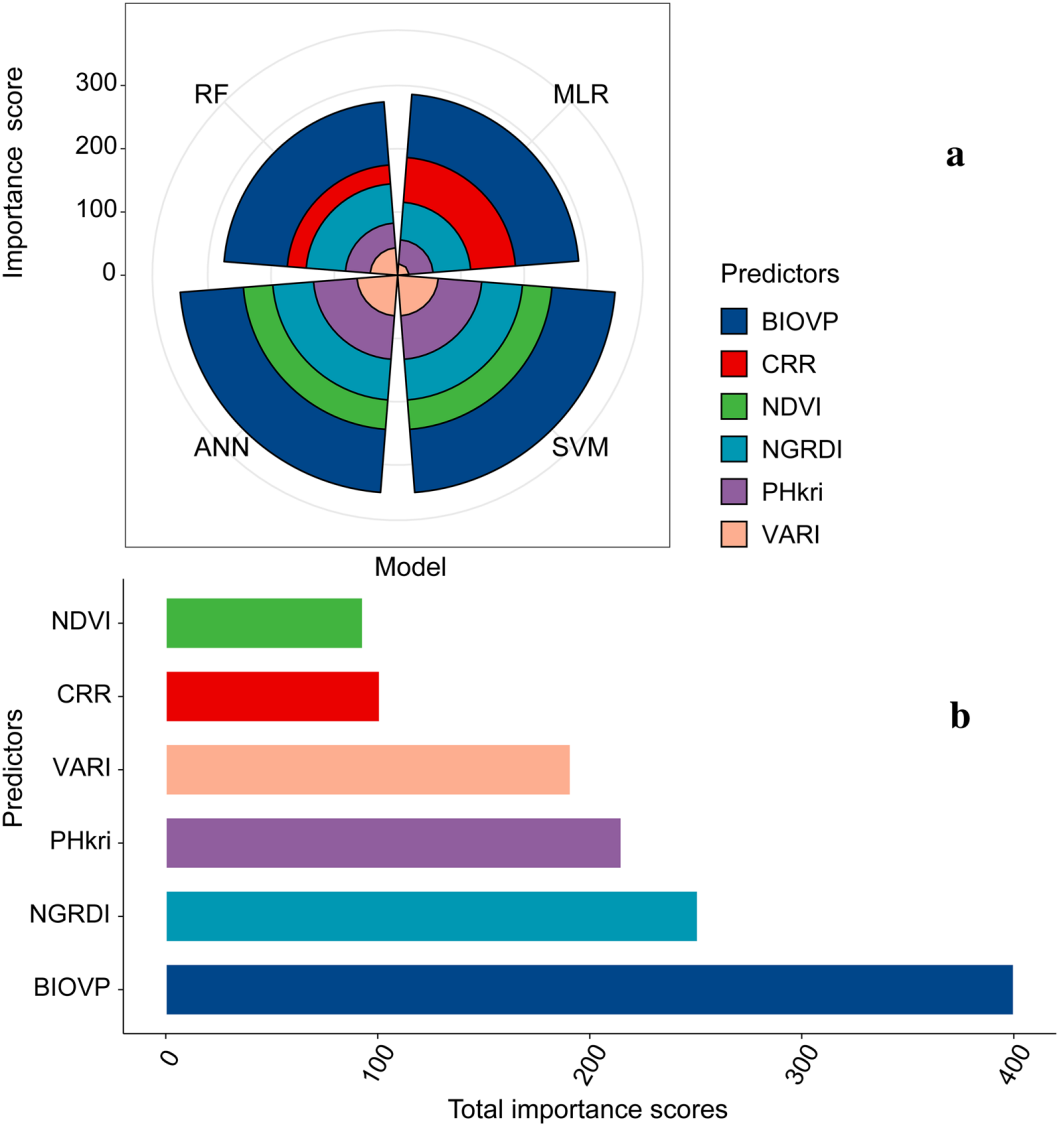
Importance scores for predictor variables. **a** The importance scores difference of predictors in different models. Predictor variables importance scores are the same in the ANN and SVM model. Because NDVI had a very small importance score in the MLR and RF models, removing it from the two models will be taken into consideration and then re-modeled. CRR can also be removed from ANN and SVM for the similar reasons. **b** The importance scores of predictors are aggregated based on four model types and are displayed on the x axis

Although the predictor variables were the same, the importance of the predictors differed between the four models (Fig. 11a). We summed up the importance scores of predictors and found that the BIOVP scores were the highest (Fig. 11b). In this study, BIOVP can retain the largest strength effect on the AGB estimate, even if different modeling strategies were used to estimate the AGB. Figure 11 also shows that plant height exerts a more direct effect than the VIs for estimating maize AGB. As a volume metric, BIOVP’s bottom area is the sum of all pixel areas imaged by vegetation. Bottom area is the product of image segmentation using vegetation index (i.e., NGRDI). Thus, BIOVP includes implicit spectral information.

**Fig. 12:**
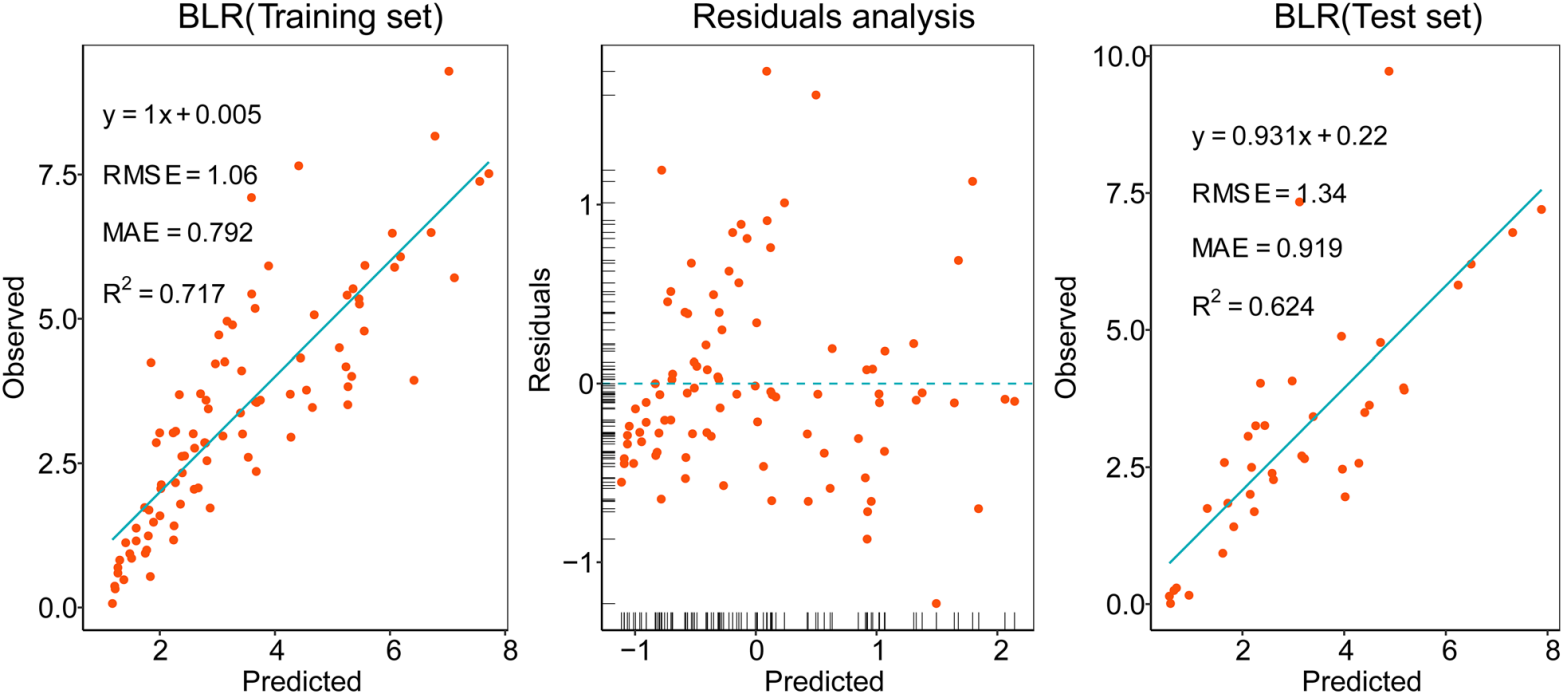
Bivariate linear-regression model based on the BIOVP. Residuals were not randomly scattered about zero with respect to the predicted values

To discover the effect of using the BIOVP to estimate AGB, we developed a bivariate linear regression (BLR) model based on the BIOVP (Fig. 12). The performance of the BLR model was even worse than the worst MLR model of the four models mentioned above. The BLR model based on the training set explained 71.7% of the variations in maize AGB, with a RMSE of 1.06 kg/m^2^ at the plot scale. The residuals-analysis plot revealed a different variance in the BLR model. Because the residuals do not appear to be randomly scattered about zero with respect to the predicted values, some predictors may have been missing from the BLR model. This result also showed that AGB estimates with a single predictor BIOVP were less effective in this study than with multiple predictors.

## Discussion

### Estimating maize height from UAV images

**Fig. 13:**
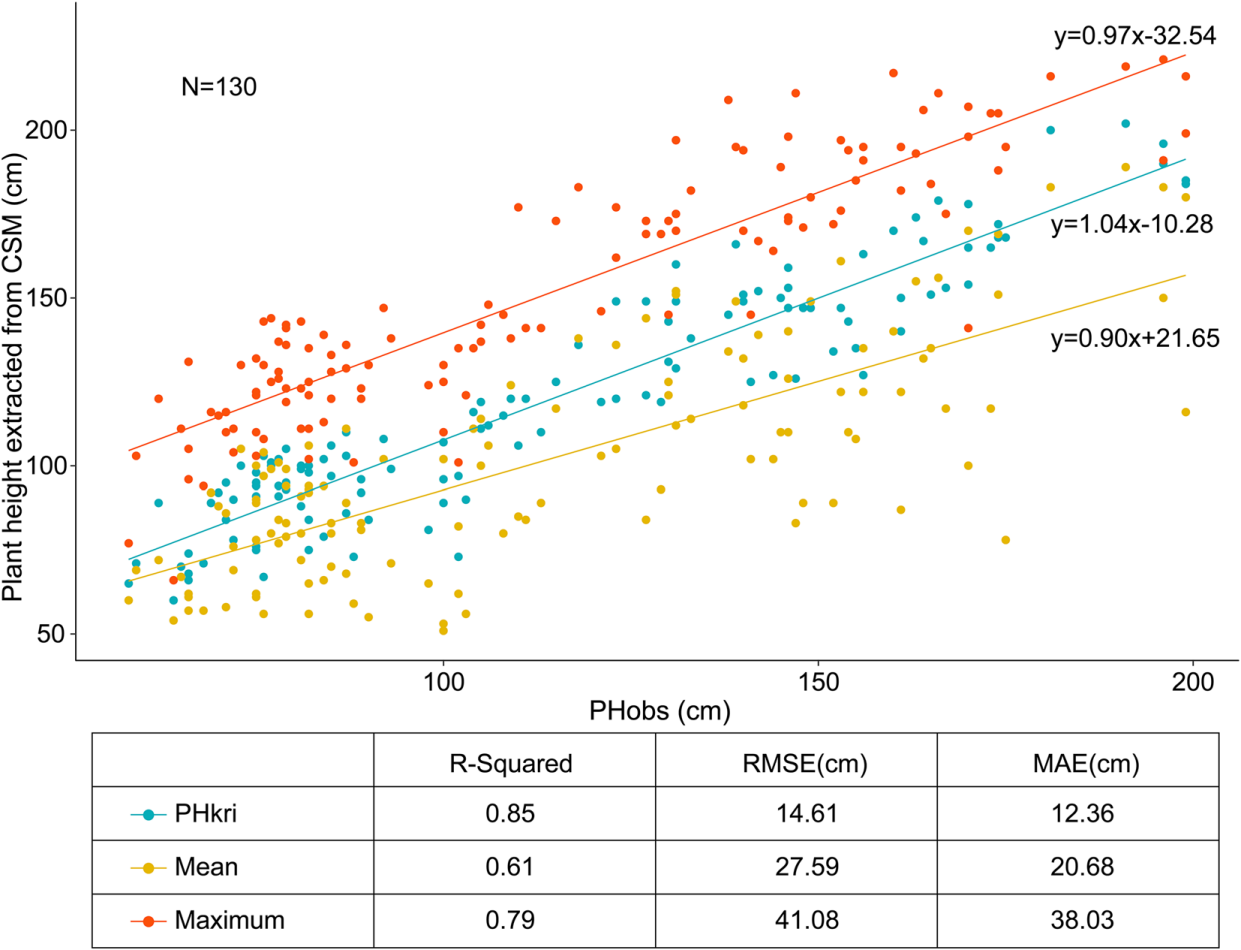
Plant height extracted from CSM versus manual ground-based measurements made with a telescopic leveling rod. PHkri, mean, and maximum are three methods to calculate plant height extracted from CSM. PHobs represents manually measured plant height

Plant height is an important crop architecture that is highly correlated with biomass yield, and several researchers have highlighted plant height to be a key contributor to biomass yield [64–66]. Because of the small planting area and low planting density in this study, relatively fewer vegetation pixels were contained in the CSM of a plot. In this scenario, if the average method was used to extract the height information from the CSM, the plant-height information would be disturbed by the soil background noise, thus causing an obvious underestimate of plant height. Previous studies have confirmed this result [1, 14, 20, 24, 25, 67, 68]. To tackle this issue, various researchers have suggested using quantiles and maximum statistics to represent plant height at the plot scale. However, these statistics were susceptible to outliers and lack explanatory power. Taking the maximum statistic as an example, from the view of digital photogrammetry technology, plant height is actually just the value of a single pixel after imaging in the CSM. Thus, the value of one pixel represents the height of multiple plants in a plot, which is not appropriate, especially in the case where the size of the plot is small and there are few plants. The most appropriate method is to calculate the plant height at the plot scale by using the pixels representing the upper leaves of multiple plants, which requires considering the spatial distribution of multiple plants in a plot. The approach we propose herein differs from previous approaches in that it considers the spatial distribution of crops and has a good mathematical interpretation. Upon comparing the plant heights calculated by the three methods with manual ground-based measurements, we found that the proposed method increased the ratio of the explained variance (R^2^ = 0.85 vs 0.61) while reducing the error (RMSE = 14.61 cm vs. 27.59 cm, MAE = 12.36 cm vs. 20.68 cm), which shows that the proposed method is feasible and effective (Fig. 13).

### Limitations and implications of study

For this study, predictor variables used for estimating maize AGB were collected rapidly and non-destructively by UAV. The UAV remote-sensing data contained uncertainties associated with multiple sources of error, which affected the accuracy of the estimate of maize AGB. Predictors measured by UAV remote sensing were all at the canopy scale and are affected by observation angle, illumination conditions, canopy structure, and leaf-morphology characteristics [69]. Because the VIs are susceptible to the confounding influences of canopy greenness and soil reflectance [70], the accumulation of maize AGB is more directly associated with changes in the physical structure of maize. When calculating the ground-based measurements of AGB on the plot scale, the growth difference between maize plants in a given plot was not considered. For destructive sampling, simply multiplying the average biomass by the number of plants may lead to systematic errors and the appearance of outliers in the data. This study thus used a small number of spectral predictor variables because of the limitation of the four-narrow-band multispectral sensor. In fact, when the UAV platform is equipped with a hyperspectral sensor, more spectral features can be used to estimate AGB [29, 71], which can reduce the collinearity and redundancy of spectral predictors that is caused by similar calculation formulas [28].

This study explored four machine-learning regression algorithms (MLR, SVM, ANN, and RF), all of which produced acceptable accuracy. The RF model yielded the best results with low error and a high ratio of the explained variance. In this study, nonlinear regression models performed significantly better than the MLR model because the former could fit the nonlinear relationship existing within the data. However, a distinct advantage of the MLR model is that it is highly interpretable [57]. The MLR model can thus be used to determine the strength of the effect that one or more predictor variable may have on a response variable by using the standardized partial regression coefficient [72].

Note also that limitations exist in the comparison of models. Because the sample size is small, the advantages and disadvantages of using different modeling strategies are not fully demonstrated. For example, the ANN model requires a lot of repeated training to obtain an optimal neural network, which requires more computer time. The inner workings of the ANN and SVM are difficult to understand, which leads to their being treated as black-box models [63]. The RF model has been applied in a wide variety of scientific areas because of its ability to resist overfitting and deal with high-dimensional data [73].

The BIOVP is a volumetric indicator for estimating maize AGB. Because image segmentation is a prerequisite for obtaining the BIOVP, this leads to an increase in the correlation between this indicator and certain spectral indices (e.g., NGRDI and VARI). Because BIOVP includes both spectral and plant-height information, both affect the accuracy of BIOVP calculations. In this study, the BIOVP was calculated by using point clouds based on digital images; point clouds based on LiDAR (light detection and ranging) are also applicable. Thus, further research is required to determine how BIOVP affects AGB estimate for different crops, scale plots, and in other scenarios.

## Conclusions

This study used multispectral and digital images collected by a UAV system to estimate maize AGB by using four machine-learning algorithms (MLR, SVM, ANN, and RF). The RF model gave the most balanced results, with low error and a high ratio of the explained variance for both the training set and the test set. We proposed herein an improved method for extracting plant height from UAV images and an indicator (BIOVP) to evaluate crop AGB. The BIOVP considers both structural and spectral information and contributes significantly to improving estimates of maize AGB. The suitability of this approach still needs to be verified for different crops and on different scales. Thus, this work suggests that structural and spectral information can be considered simultaneously rather than separately when estimating biophysical crop parameters.

## Additional files

**Additional file 1.** Method for calculating total error of estimating GCPs location in UAV images.

**Additional file 2.** A schematic illustration for explaining the concepts of BIOVP and PHkri.

**Additional file 3.** Running R scripts for machine learning modeling and diagnostic plots.

## Abbreviations

AGB: above-ground biomass
AGL: flight altitude above ground level
ANN: artificial neural network
ANOVA: analysis of variance
AOI: area of interest
BIOVP: a volume metric used to estimate crop biomass within a plot
BLR: bivariate linear regression
CRR: canopy elevation relief ratio
CIgreen: chlorophyll index green
CIrededge: chlorophyll index rededge
CVI: chlorophyll vegetation index
CSM: crop surface model
DSMs: digital surface models
DEM: digital elevation model
ExG: excess green index
GLI: green leaf index
MAE: mean absolute error
GSD: ground sampling distance
MLR: multiple linear regression
NGRDI: normalized green–red difference index
NDRE: normalized difference red-edge
NDVI: normalized difference vegetation index
PHobs: plant height measured by manpower
PHkri: plant height calculated by using a Kriging interpolation
RF: random forest
R^2^: coefficient of determination
RMSE: root mean square error
RVI: ratio vegetation index (also called simple ratio)
SVM: support vector machine
UAV: unmanned aerial vehicle
VIs: vegetation indices
VARI: visible atmospherically resistant index
WDRVI: wide dynamic range vegetation index
