# Supplementary material for: Modeling maize above-ground biomass based on machine learning approaches using UAV remote-sensing data
Source: Plant Methods. 2019 Feb 4;15:10. doi: 10.1186/s13007-019-0394-z (PMC6360736; doi:10.1186/s13007-019-0394-z)
Supplement: Supplementary file 1 — Additional file 1. Method for calculating total error of estimating GCPs location in UAV images. [file 13007_2019_394_MOESM1_ESM.doc]

**Additional file 1: Calculate error of the GCPs**

Differential Global Positioning System (DGPS) provides millimeter positioning accuracy. So the result of using DGPS measurement is regarded as the real world positioning coordinate, that is, ground truth of GCPs. These errors are the three dimensional coordinate differences of GCPs between **ground truth** and **the position in the UAV images** (estimate value). For example, E1-E3 present the position of three GCPs in the UAV images as shown in the figure below.


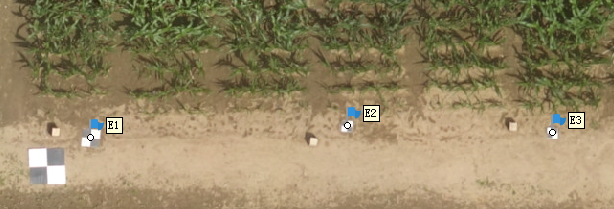

$$\text{Total error}=\sqrt{\frac{1}{N}\left(\sum_{i=1}^{N} \left(X_{i}-\hat{X}_{i}\right)^{2}\text{+}\sum_{i=1}^{N} \left(Y_{i}-\hat{Y}_{i}\right)^{2}\text{+}\sum_{i=1}^{N} \left(Z_{i}-\hat{Z}_{i}\right)^{2}\right)}$$

$$\text{X error}=\sqrt{\frac{1}{N}\left(\sum_{i=1}^{N} \left(X_{i}-\hat{X}_{i}\right)^{2}\right)}$$

$$\text{Y error}=\sqrt{\frac{1}{N}\left(\sum_{i=1}^{N} \left(Y_{i}-\hat{Y}_{i}\right)^{2}\right)}$$

$$\text{Z error}=\sqrt{\frac{1}{N}\left(\sum_{i=1}^{N} \left(Z_{i}-\hat{Z}_{i}\right)^{2}\right)}$$

where *N* is the total sample size (*N*=1,2,3…16), $X_{i}$,$Y_{i}$and $Z_{i}$ is the ith ground truth of GCP, $\hat{X}_{i}$,$\hat{Y}_{i}$ and $\hat{Z}_{i}$ is the ith estimate value of GCP.
