# Supplementary material for: Modeling maize above-ground biomass based on machine learning approaches using UAV remote-sensing data
Source: Plant Methods. 2019 Feb 4;15:10. doi: 10.1186/s13007-019-0394-z (PMC6360736; doi:10.1186/s13007-019-0394-z)
Supplement: Supplementary file 2 — Additional file 2. A schematic illustration for explaining the concepts of BIOVP and PHkri. [file 13007_2019_394_MOESM2_ESM.doc]

**Additional file 2:** Schematic illustration of BIOVP and PHkri


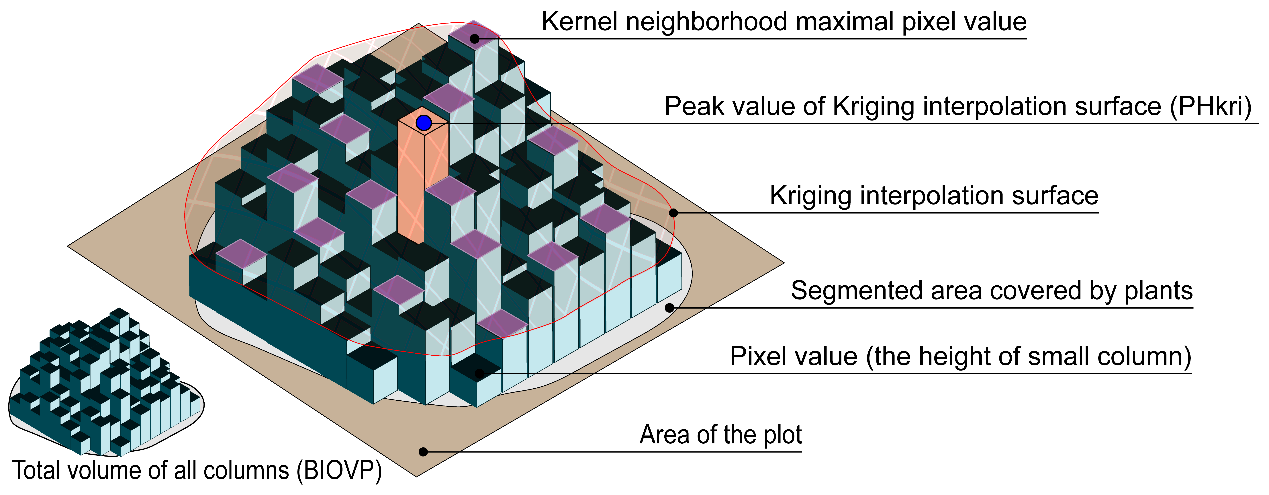


- The height of small column represents pixel value, which indicates plant height information.
- The curve surface with red border is interpolated surface.
- The segmentation area covered the plants was obtained by using spectral vegetation index operation.
- Interpolated surface was generated based on kernel neighborhood maximal pixel value by using space Kriging interpolation method in the ArcMap.
- PHkri is the peak point of this interpolated surface. Clearly, PHkri is the result of interpolation operation, and represents the plant height of maize population at the plot scale.
- BIOVP is the total volume of all small column. As a volume metric, BIOVP's bottom area is the sum of all pixel areas imaged by vegetation. Bottom area is the product of image segmentation using vegetation index (i.e., NGRDI). Thus, BIOVP implies spectral information.
