# Supplementary material for: Modeling maize above-ground biomass based on machine learning approaches using UAV remote-sensing data
Source: Plant Methods. 2019 Feb 4;15:10. doi: 10.1186/s13007-019-0394-z (PMC6360736; doi:10.1186/s13007-019-0394-z)
Supplement: Supplementary file 3 — Additional file 3. Running R scripts for machine learning modeling and diagnostic plots. [file 13007_2019_394_MOESM3_ESM.doc]

**Additional file 3: Running R Scripts for machine learning modeling and diagnostic chart（MLR,ANN,RF,SVM）**

Data format(.csv)


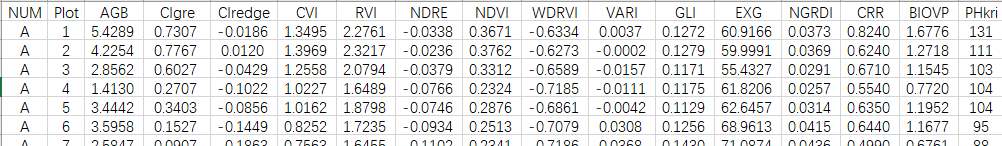


**## Load package**

library(caret)

library(ggplot2)

library(ggpubr)

**## Reading data and standardization**

biodataset <- read.csv(file.choose(), header=T)

pp_boxcox <-preProcess(biodataset[-1],method=c("BoxCox","center","scale"))

AllFeatures<-predict(pp_boxcox,biodataset[-1])

**# Select predictors using RFE algorithm**

rfeCtrl<-rfeControl(method="repeatedcv",

repeats=10,number=10,

verbos=TRUE,

functions=rfFuncs)

set.seed(100097)

rfRFE<-rfe(x=AllFeatures[,2:15],

y=AllFeatures$AGB,

size=c(2:20),

savePredictions = TRUE,

rfeControl=rfeCtrl

)

rfRFE

#The top 5 variables (out of 6):

BIOVP, PHkri, NGRDI, VARI, CRR

BioFeatures<-select(biodataset,NUM,AGB,BIOVP,NDVI,CRR,NGRDI,VARI,PHkri)

set.seed(100097)

**#training set by stratified sampling**

trainingRows <-createDataPartition(BioFeatures$NUM,p=0.70,list=FALSE)

trainSets<-BioFeatures[trainingRows,]

#Test set by stratified sampling

testSets<-BioFeatures[-trainingRows,]

#using repeated 10 fold cross validation

ctrKFCV<-trainControl(method="repeatedcv",repeats=10,number=10)

# MLR model

set.seed(100)

lmModel<-train(AGB~., data=trainSets,

method = "lm",

trControl = ctrKFCV)

lmModel

#predict

lmTrainY <- predict(lmModel)

#Averaged Neural Network Model

# Grid search and tuning parameters

nnetGrid<-expand.grid(decay = c(0.001, 0.01, 0.1),

size = c(1:6),

bag = FALSE)

set.seed(100)

nnetModel <-train(AGB~., data=trainSets,

method = "avNNet",

tuneGrid=nnetGrid,

linout=TRUE,

trace=TRUE,

maxit=500,

trControl = ctrKFCV)

nnetModel

#The final values used for the model were size = 2, decay = 0.1 and bag = FALSE.

#predict

nnetTestY <- predict(nnetModel,testSets)

# SVM model

set.seed(100)

svmGrid <- expand.grid(sigma = seq(0.01,0.2,by=0.001),C = 2^(-2:11))

svmRModel <- train(AGB~., data=trainSets,

method = "svmRadialSigma",

tuneLength = 10,

tuneGrid = svmGrid,

trace = TRUE,

metric="RMSE",

trControl = ctrKFCV

)

svmRModel

#The final values used for the model were sigma = 0.0254 and C = 4.

#predict

svmTestY <- predict(svmRModel,testSets)

set.seed(100)

rfModel<-train(AGB~., data=trainSets,

method="rf",

tuneLength=6,

n.trees=1000,

importance=TRUE,

trControl=ctrKFCV)

rfModel

#RMSE was used to select the optimal model using the smallest value.

#The final value used for the model was mtry = 2.

#predict

rfTestY <- predict(rfModel,testSets)

# importance score of predictors

varImp(rfModel)

rf variable importance

Overall

BIOVP 100.00

NGRDI 62.18

VARI 43.50

PHkri 39.14

CRR 29.99

NDVI 0.00

varImp(nnetModel)

Overall

BIOVP 100.00

PHkri 68.84

NGRDI 64.52

VARI 64.40

NDVI 46.63

CRR 0.00

varImp(lmModel)

varImp(svmRModel )

# scatter plot for MLR model

# in training set

lmtrScatPlo<-ggplot(data=lmTrPlotSets, aes(x=pred, y=obs)) +

geom_point(colour="#FC4E07")+stat_smooth(method="lm",se=FALSE,colour="#00AFBB")+labs(x="Predicted",y="Observed",title="MLR(Training set)")+ theme_bw()+theme(title=element_text(size=15,color="black",hjust=0.5),

axis.title.x=element_text(size=15,hjust=0.5),

axis.title.y=element_text(size=15,hjust=0.5), axis.text.x=element_text(size=15,color="black"),

axis.text.y=element_text(size=15,color="black"))+

theme(plot.title = element_text(hjust = 0.5))+

theme(panel.grid.major = element_blank(),panel.grid.minor = element_blank(),axis.line = element_line(colour = "black"))+

annotate("text",x=1,y=8.5,,parse=TRUE,label="y==1.001*x+0.003",size=5,hjust=0)+

annotate("text",x=1,y=7.5,,parse=TRUE,label="RMSE==0.986",size=5,hjust=0)+

annotate("text",x=1,y=6.5,,parse=TRUE,label="MAE==0.714",size=5,hjust=0)+

annotate("text",x=1,y=5.5,,parse=TRUE,label="R^2==0.757",size=5,hjust=0)

lmtrScatPlo

#In test set

lmteScatPlo<-ggplot(data=lmTePlotSets, aes(x=pred, y=obs)) +

geom_point(colour="#FC4E07")+stat_smooth(method="lm",se=FALSE,colour="#00AFBB")+

labs(x="Predicted",y="Observed",title="MLR(Test set)")+theme_bw()+

theme(title=element_text(size=15,color="black",hjust=0.5),

axis.title.x=element_text(size=15,hjust=0.5),

axis.title.y=element_text(size=15,hjust=0.5), axis.text.x=element_text(size=15,color="black"),

axis.text.y=element_text(size=15,color="black"))

theme(plot.title = element_text(hjust = 0.5))+

theme(panel.grid.major = element_blank(),panel.grid.minor = element_blank(),axis.line = element_line(colour = "black"))+

annotate("text",x=1,y=8.5,,parse=TRUE,label="y==0.914*x+0.275",size=5,hjust=0)+

annotate("text",x=1,y=7.5,,parse=TRUE,label="RMSE==1.278",size=5,hjust=0)+

annotate("text",x=1,y=6.5,,parse=TRUE,label="MAE==0.876",size=5,hjust=0)+

annotate("text",x=1,y=5.5,,parse=TRUE,label="R^2==0.661",size=5,hjust=0)

lmteScatPlo

#Residual analysis chart

lmResiPlotSets<-data.frame(Predicted=lmTrainY,Residuals=lmModel$finalModel$residuals)

lmResiPlo<-ggplot(data=lmResiPlotSets, aes(x=Predicted, y=Residuals)) +

geom_point(colour="#FC4E07")+ geom_hline(yintercept=0,linetype="dashed",colour="#00AFBB")+

#Marginal rug line

geom_rug(position="jitter",size=0.2)+ labs(title="Residuals analysis")+

theme_bw()+

theme(title=element_text(size=15,color="black",hjust=0.5),

axis.title.x=element_text(size=15,hjust=0.5),

axis.title.y=element_text(size=15,hjust=0.5), axis.text.x=element_text(size=15,color="black"),

axis.text.y=element_text(size=15,color="black"))+

theme(plot.title = element_text(hjust = 0.5))+

theme(panel.grid.major = element_blank(),panel.grid.minor = element_blank(),axis.line = element_line(colour = "black"))

lmResiPlo

#Combinatorial graph

grid.arrange(lmtrScatPlo,lmResiPlo,lmteScatPlo,ncol=3)
